# Phytochemical Profiles and Antimicrobial Activities of *Allium cepa* Red cv. and *A. sativum* Subjected to Different Drying Methods: A Comparative MS-Based Metabolomics

**DOI:** 10.3390/molecules22050761

**Published:** 2017-05-08

**Authors:** Mohamed A. Farag, Sara E. Ali, Rashad H. Hodaya, Hesham R. El-Seedi, Haider N. Sultani, Annegret Laub, Tarek F. Eissa, Fouad O. F. Abou-Zaid, Ludger A. Wessjohann

**Affiliations:** 1 Pharmacognosy department, College of Pharmacy, Cairo University, Kasr el Aini St., P.B. 11562 Cairo, Egypt; 2 Department of Pharmaceutical Biology, Faculty of Pharmacy & Biotechnology, The German University in Cairo, P.B. 11835 Cairo, Egypt; saraezz16512@gmail.com; 3 Plant production Department, Desert Research Center, P.B. 11714 Cairo, Egypt; balance2000@hotmail.com (R.H.H.); foad-omar@hotmail.com (F.O.F.A.-Z.); 4 Division of Pharmacognosy, Department of Medicinal Chemistry, Uppsala University, Box 574, SE-75 123 Uppsala, Sweden; hesham.el-seedi@fkog.uu.se; 5 Department of Chemistry, Faculty of Science, El-Menoufia University, 32512 Shebin El-Kom, Egypt; 6 Leibniz Institute of Plant Biochemistry, Dept. Bioorganic Chemistry, Weinberg 3, D-06120 Halle (Saale), Germany; haidersoltani@yahoo.com (H.N.S.); Annegret.Laub@ipb-halle.de (A.L.); 7 Pharmacognosy Department, College of Pharmacy, Modern Science and Arts University, P.B. 12566, Cairo, Egypt; eissatarek@hotmail.com

**Keywords:** garlic, onion, metabolomics, drying, organosulphur, flavonoids, anti-microbial

## Abstract

Plants of the *Allium* genus produce sulphur compounds that give them a characteristic (alliaceous) flavour and mediate for their medicinal use. In this study, the chemical composition and antimicrobial properties of *Allium cepa* red cv. and *A. sativum* in the context of three different drying processes were assessed using metabolomics. Bulbs were dried using either microwave, air drying, or freeze drying and further subjected to chemical analysis of their composition of volatile and non-volatile metabolites. Volatiles were collected using solid phase micro-extraction (SPME) coupled to gas chromatography–mass spectrometry (GC/MS) with 42 identified volatiles including 30 sulphur compounds, four nitriles, three aromatics, and three esters. Profiling of the polar non-volatile metabolites via ultra-performance liquid chromatography coupled to high resolution MS (UPLC/MS) annotated 51 metabolites including dipeptides, flavonoids, phenolic acids, and fatty acids. Major peaks in GC/MS or UPLC/MS contributing to the discrimination between *A. sativum* and *A. cepa* red cv. were assigned to sulphur compounds and flavonoids. Whereas sulphur conjugates amounted to the major forms in *A. sativum*, flavonoids predominated in the chemical composition of *A. cepa* red cv. With regard to drying impact on *Allium* metabolites, notable and clear separations among specimens were revealed using principal component analysis (PCA). The PCA scores plot of the UPLC/MS dataset showed closer metabolite composition of microwave dried specimens to freeze dried ones, and distant from air dried bulbs, observed in both *A. cepa* and *A. sativum*. Compared to GC/MS, the UPLC/MS derived PCA model was more consistent and better in assessing the impact of drying on *Allium* metabolism. A phthalate derivative was found exclusively in a commercial garlic preparation via GC/MS, of yet unknown origin. The freeze dried samples of both *Allium* species exhibited stronger antimicrobial activities compared to dried specimens with *A. sativum* being in general more active than *A. cepa* red cv.

## 1. Introduction

Onion (*Allium cepa* L.) and garlic (*A. sativum* L.) are among the oldest cultivated plants, used for culinary purposes in addition to their therapeutic effects [1]. *Allium* species present a rich source of phytonutrients of potential health benefits for treatment of diabetes type 2, coronary heart disease, obesity, hypercholesterolemia, hypertension, cataract, and disturbances of the gastrointestinal tract. Interest in garlic cancer chemopreventive effect is based on epidemiological studies showing a decrease of gastric cancer risk proportional to the increase of garlic intake. Such evidence has been related to the ability of garlic to reduce nitrite levels in the gastric tract [1,2].

Most of *Allium* biological effects are related to the sulphur-containing compounds, “thiosulphinates”, typical of *Allium* and responsible for its characteristic pungent aroma and taste. Nevertheless, these metabolites are relatively unstable which warrants the development of analytical methods with which changes in their structure can be monitored i.e., in response to processing methods. Compared to sulphur compounds, other constituent viz., saponins and flavonoids found in *Allium* are more stable to cooking and storage conditions [2]. *Allium* are consumed either as raw vegetable (fresh leaves or dried cloves), or after processing in the form of oil, extract or powder. Pronounced differences in the chemical composition and content of their bioactive compounds are observed during processing. Recently, the impact of processing methods on functional foods chemical composition and quality has been under increasing scrutiny [1].

Metabolomics is increasingly employed to gain insight into the chemical composition of biological materials. At present, ultra performance liquid chromatography-mass spectrometry (UPLC/MS) and gas chromatography mass spectrometry (GC/MS) are two efficient platforms mostly employed to resolve the complex plant metabolome. Whereas UPLC/MS favours the analysis of non-volatile polar or semi-polar metabolites, GC/MS is suited for the analysis of volatiles, which define the aroma of a plant. GC/MS indeed provides complementary data to UPLC/MS analysis for defining *Allium* aroma, as volatiles release arises from the breakage of the non-volatile precursors i.e., glycosides only detected with UPLC/MS [3].

The present study aims to assess the impact of three drying methods *viz*., shade-drying, freeze drying, and microwave-drying, on the chemical composition of onion and garlic cloves as analysed via solid-phase microextraction (SPME) coupled to (GC/MS) and in parallel to UPLC/MS. Headspace SPME is a relatively novel technique used for volatiles extraction and has been found to be superior to other methods, being solvent free and involving no heat application. One powerful feature of SPME volatiles sampling lies in preserving the true aroma without development of artifact that might be generated with heating as in the case of steam distillation [4]. Considering the complexity of spectral data, multivariate data analyses were employed to classify samples in an untargeted manner. Analysed samples included *A. sativum* and *A. cepa* red cv. bulbs dried using three methodologies in addition to a commercial garlic product containing dried garlic powder. Further, considering that the antimicrobial activity of *Allium* species is a well-recognized effect [5], it was of interest to determine how these different drying methods can impact such effect in relation to changes in metabolites composition as monitored using metabolomics.

## 2. Results

### 2.1. Identification of Allium Species Volatiles via SPME-GC/MS

SPME was employed to analyze *Allium* headspace volatiles and then the trapped volatiles were subsequently analysed via GC/MS [6]. The biological variance within each specimen was assessed using three indpendent biological replicates, subjected to the same extraction and analysis conditions. Volatiles analysis using SPME led to the detection of 42 volatile components belonging to 30 sulphur containing volatiles, four nitriles, two nitrogenous volatiles, three aromatics and three esters. Representative GC/MS chromatogram of freeze dried *A. sativum* and *A. cepa* red cv. is displayed in (Supplementary materials Figure S1). The identity, retention time (r.t.), retention index (RI) and mass-to-charge ratio (*m*/*z*) of these compounds are shown in Table 1. Volatiles belonged to various classes including mostly sulphur and non-sulphur. Sulphur compounds constituted the most dominant volatile class which was found more enriched in *A. sativum* with a relative percentile of (99.8%) versus (83%) in *A. cepa*. Major identified sulphur volatiles included diallyl disulphide (45–99%) in *A. sativum* versus allyl methyl trisulphide (13–20%) in *A. cepa*. In the current study, several sulphur rearrangement products i.e., allyl compounds identified in peaks M1, M6, M10, M14, M16, M28, M29, M32, and M36 along with a cyclic, 3-vinyl-1,2-dithiacyclohex-5-ene (M21) were detected in *A. sativum,* and likely to have been formed at the high temperature of the GC/MS injection port. Short chain sulphur molecules exemplified in dimethyl trisulphide (M3), dimethyl tetrasulphide (M23) and dipropyl trisulphide (M30) were identified mostly in *A. cepa,* reported as degradation products in cooked *Allium*. With regard to sulphur compounds abundance, *A. sativum* was found much more enriched in this volatile class compared to *A. cepa* in all examined specimens. Next to sulphur containing compounds, esters amounted to the major volatile form especially in the commercially dried garlic product “Tomex” represented by diethylphthalate at ca. 99% of its volatile blend. A few aromatics were identifed almost exclusively in *A. sativum* including cuminaldehyde (M25) and 3-isopropylbenzaldehyde (M26), although at trace levels.

### 2.2. Multivariate Data Analysis of Allium Species Analysed via SPME-GC/MS

**Figure 1:**
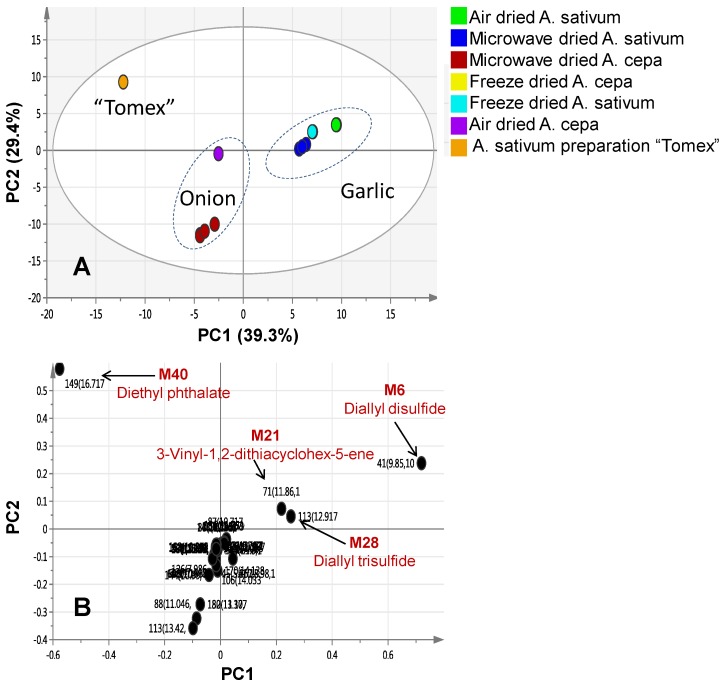
Solid-phase microextraction coupled to gas chromatography mass spectrometry) (SPME-GC/MS) based principal component analysis (PCA) of fresh and dried *A. sativum* and *A. cepa* red cv. specimens. (**A**) Score plot of PC1 and PC2 scores; (**B**) Loading plot for PC1 components contributing peaks and their assignments, with each metabolite denoted by its mass/r.t. (min) value: M6; diallyl disulphide, M21; 3-Vinyl-1,2-dithiacyclohex-5-ene, M28; diallyl trisulphide and M40; diethyl phthalate. Peak numbering follows that listed in (Table 1) for volatiles identification using SPME-GC/MS.

To better visualize the subtle similarities and differences either between *A. sativum* and *A. cepa* red cv. or in response to the different drying methods, multivariate data analyses were employed. Principal component analysis (PCA) is an unsupervised clustering process for identifying patterns in data, via reducing the number of dimensions. It can define a limited number of principal components which describe independent variation in the results [3]. In the present study, PCA was first applied to classify the different forms of onion and garlic with respect to their chemical composition and to determine whether a genotype effect could overcome the drying method adopted herein. A commercial dried powder garlic film-coated tablet was also included for comparison. The PCA score plot was able to readily discriminate between both species regardless of the drying method, with garlic samples clustering separately on the positive score values of PC1, whereas onion specimens were positioned at the negative side of PC1 (Figure 1A).

The first two components PC1 and PC2 explained 39.3% and 29.4% of the total variance, respectively. Separation based on the type of processing method could not be observed from PCA, suggesting that species-based separation predominates over drying method impact. The corresponding loading plot of PC1 describes the most discriminatory metabolites in each group leading to such segregation and revealing that garlic samples encompassed higher diallyl disulphide, diallyl trisulphide, and 3-vinyl-1,2-dithiacyclohex-5-ene levels as compared to onion samples (Figure 1B).

Interestingly, garlic commercial tablets “Tomex” failed to group with other garlic specimens. Examination of the loading plot revealed its enrichment in diethyl phthalate, which was absent from other garlic samples and accounting for its dispersal (Figure 1B).

**Figure 2:**
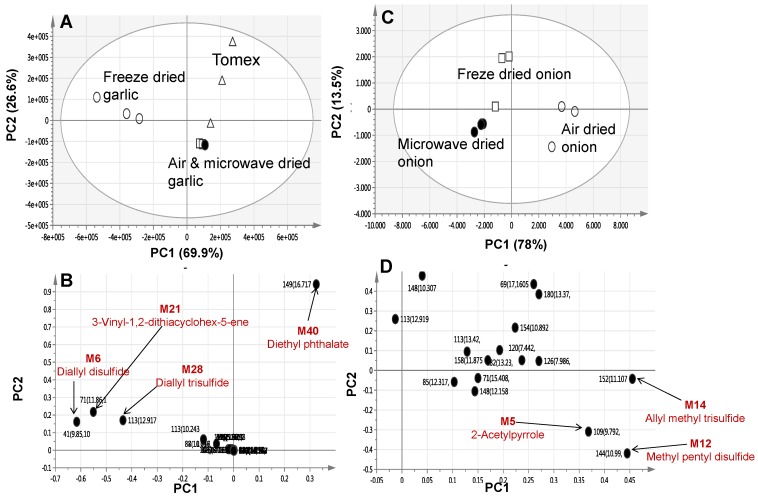
GC/MS based PCA score plot derived from modelling drying effect on *A. sativum* (**A**); *A. cepa* red cv. (**C**) one at a time separately to assess the effect of drying on metabolite composition (*n* = 3). The loading plot from *A. sativum* (**B**) and *A. cepa* red cv; (**D**) shows the most variant masses detected using GC/MS and contributing to the samples segregation. Volatiles are denoted with m/z/retention time (sec) pair and identifications are discussed in text. M6—diallyl-disulphide, M21—3-Vinyl-1,2-dithiacyclohex-5-ene, M28—diallyl trisulphide, and M40—diethyl phthalate. Peak numbering follows those listed in (Table 1) for volatiles identification using SPME-GC/MS.

Considering our interest in investigating the possible influence of the three drying methods for each *Allium* species, a second PCA trial model was constructed for individual specimens of each species. The garlic model score plot (PC1 = 69.9% and PC2 = 26.6%) showed that freeze dried garlic was clearly separated from shade air-dried and microwave-dried garlic along PC1, suggesting degradation of sulphur compounds to a similar extent in both shade air-dried and microwave-dried specimens (Figure 2A). The corresponding loading plot revealed high diallyl disulphide, diallyl trisulphide, and 3-vinyl-1,2-dithiacyclohex-5-ene levels in freeze-dried garlic samples (Figure 2B). In agreement with the first PCA results (Figure 1B), distant clustering of samples belonging to commercial garlic preparation “Tomex” was due to its enrichment with diethyl phthalate.

Phthalic acid esters (PAEs) are employed in polymer materials as a plasticizer commonly found in organic solvents not of high grade and our data suggest that they might have originated during the manufacturing process [7]. In spite of the clear separation observed in PCA for freeze dried garlic samples, a supervised method as orthogonal projection to latent structures-discriminant analysis (OPLS-DA) was further applied to help identify metabolites indicative of each processing method. The OPLS model was evaluated by the two parameters, Q^2^Y and R^2^X, where R^2^X is used to quantify the goodness-of-fit, whereas Q^2^Y is employed to assess the predictability of the model [8]. An OPLS-DA model was constructed by modelling freeze dried garlic against air-dried and microwave-dried samples grouped together in another class group with clear separation between freeze dried and other drying methods (Supplement Figure S2A). The S-loading plot of the OPLS-DA model revealed for the freeze-dried garlic samples enrichment in diallyl disulphide, diallyl trisulphide, and 3-vinyl-1,2-dithiacyclohex-5-ene (Figure S2B) and concurring the PCA results (Figure 2B).

Likewise, a PCA model was constructed for freeze-dried, shade air-dried, and microwave-dried onion samples with variance explained by PC1 = 78% and PC2 = 13.5%. Microwave-dried onion samples were clustered closely with freeze-dried samples (negative score values), whereas air-dried samples were positioned on the other side with positive score values of PC1 (Figure 2C). The PCA loading plot revealed that 2-acetylpyrrole, methyl pentyl disulphide and allyl methyl trisulphide were the most representative metabolites detected in the air-dried samples (Figure 2D).

Discrepancy between the *Allium* species drying model results suggest that drying affects *Allium* sp. in different ways as monitored via GC/MS. To confirm such a hypothesis, a different analytical platform was adopted for *Allium* metabolites profiling. Heat applied during evaporation of *Allium* sulphur compounds in GC/MS could have led to degradation of its native thiosulphinates [9]. Indeed, thermal instability of thiosulphinates warrants the utilization of a less artifact prone methodology viz. ultra performance liquid chromatography (HPLC) coupled to MS. Compared to GC/MS, UPLC/MS is more suited for the analysis of non-volatile polar constituent viz., glycosides, peptides found in *Allium* [2].

### 2.3. Identification of Allium Species Non-Volatile Metabolites via UPLC/PDA/qTOF-MS

**Figure 3:**
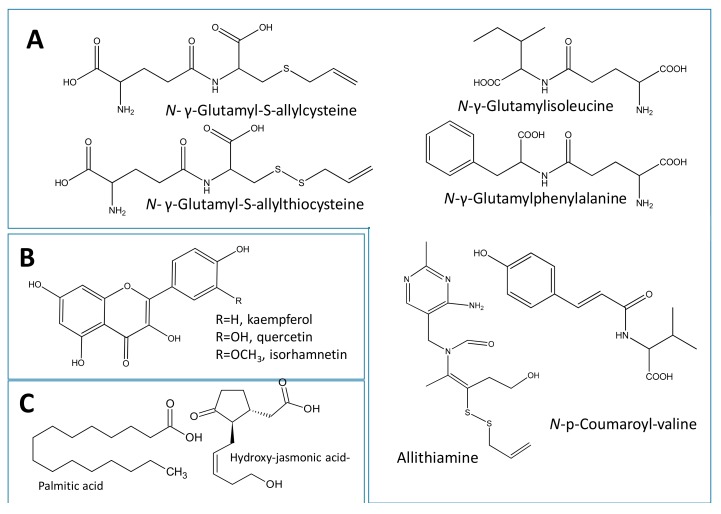
Examples of natural product classes reported and detected in genus *Allium* via UPLC/MS with selected compound(s) discussed in the manuscript. (**A**) Peptides and amino acids; (**B**) flavonols and (**C**) fatty acids/oxylipids.

Phytoconstituents of *A. cepa* red cv. and *A. sativum* were analysed via reversed-phase UPLC/PDA/ESI-qTOF-MS, using a gradient mobile phase consisting of acetonitrile and formic acid. Complete elution of metabolites was achieved within a short time (ca. 20 min). UPLC-qTOF-MS using electrospray ionization (UPLC-ESI-MS) is regarded as a particularly well accepted platform for untargeted plant metabolite profiling [10]. The technology has been recently applied to assess the effect of fermentation on organosulphur compounds in garlic [11]. In the current study, a total of 51 metabolites were detected as listed in Table 2. Metabolites assignment was made by comparing retention times, UV-vis spectra, MS data (accurate mass, isotopic distribution and fragmentation pattern) with the reported literature of *Allium* and by searching the phytochemical dictionary of natural products database. Metabolites belonged to various classes including sulphur and non-sulphur containing peptides, flavonoids, phenolic acids, and fatty acids (Figure 3). Metabolites were eluted in a decreasing order of polarity, whereby dipeptides and phenolic acids appeared first in the chromatogram followed by flavonoid di- and monoglucosides, aglycones, and finally fatty acids (Figure S3).

#### 2.3.1. Identification of Dipeptides and Amino Acid Conjugates

The organosulphur dipeptides in *Allium* are of special value considering that they are the mediators of its medicinal use and organoleptic characters. Particularly, garlic is enriched in γ-glutamyl peptides and sulfoxides [12]. The main organosulphur compounds detected in garlic were *N*-γ-glutamyl-*S*-allylcysteine, *N*-γ-glutamyl-*S*-allylthiocysteine, allithiamine and *N*-hexosyl-*N*-γ-glutamyl-*S*-allylcysteine identified in peaks L6/10, L8/15, L13, and L5.

*N*-γ-glutamyl-*S*-allylcysteine isomers were identified in peaks L6 and L10 (*m*/*z* 289.0873, [M − H]^−^, C_11_H_17_N_2_O_5_S) whereas two isomers of *N*-γ-glutamyl-*S*-allylthiocysteine were assigned in peaks L8 and L15 (*m*/*z* 321.0612, [M − H]^−^, C_11_H_17_N_2_O_5_S_2_). Lower fragment masses at *m*/*z* 128 attributed to a sequential loss of glutamine residue along the amide linkage in addition to loss of water (−18 amu) were characteristic in these dipeptides (Supplement Figure S4A,B). Additionally, a fragment appearing at *m*/*z* 249 due to the breakage of the allyl sulphur moiety was evident in peaks L8 and L15 (Figure S4B).

Allithiamine, or thiamine allyl disulphide, a lipid-soluble form of vitamin B1 which occurs in garlic [13] was detected in peak L13 (*m*/*z* 353.0285, [M − H]^−^, C_15_H_21_N_4_O_2_S_2_). Glycosidic conjugate of *N*-γ-glutamyl-*S*-allylcysteine was assigned to peak L5 (*m*/*z* 451.1401, [M − H]^−^, C_17_H_27_O_10_N_2_S) exhibiting the neutral loss of 162 amu for hexose moiety (Supplement Figure S4C). Similar hexose loss was detected in the two non-sulphur containing dipeptides first time reported in *Allium* (peak L9) [*m*/*z* 421.182, [M − H]^−^, C_17_H_29_N_2_O_10_] and (peak L12) [*m*/*z* 455.1666, [M − H]^−^, C_20_H_27_N_2_O_10_] and assigned as *N*-hexosyl-γ-glutamylisoleucine and *N*-hexosyl-glutamylphenylalanine, respectively (Supplement Figure S4D,E). The two corresponding parent glutamine dipeptides (L7 and L11) were identified by high resolution MS with [M − H]^−^ of 259.1298, and 293.1135, respectively. Lower *m*/*z* fragment ions at 128 in both peaks was attributed to glutamine loss along the amide linkage and assigned as *N*-hexosyl-γ-glutamylisoleucine (L7) and *N*-γ-glutamylphenylalanine (L11) (Supplement Figure S4F,G). Loss of 80 amu for sulphate moiety was evident in an unknown peak L3 with a molecular ion of *m*/*z* 337.1711, (C_18_H_27_O_3_NS) (Supplement Figure S4H). A new nitrile was also tentatively annotated as a simmondisin derivative (L4) from its molecular ion at *m*/*z* 337.1711, (C_18_H_27_O_3_NS)^−^. Two novel acylated peptides were detected in peaks L28 and L30 (*m*/*z* 262.1089, [M − H]^−^, C_14_H_16_NO_4_) containing valine as revealed from tandem MS spectra and identified as *N*-*p*-coumaroyl-valine isomers.

#### 2.3.2. Identification of Flavonoids

Medicinal plants rich in polyphenols can retard the oxidative degradation of lipids and improve the quality and nutritional value of food [14]. Garlic and particularly onion are considered one of the richest sources of phenolic compounds i.e., flavonoids [15]. Photodiode array inspection of peaks assisted in capturing an overview of their main flavonoid subclass. Flavonol glycosides constituted the most abundant class mostly enriched in *A. cepa* red cv. extracts as revealed from their UV max near 270 nm and a second maximum (345–360 nm). MS/MS analysis led to the identification of the aglycone (Ag) moiety, where the sugar type in *O*-glycosides could be determined from the respective loss of 162, 146, and 132 amu corresponding to hexose, deoxyhexose, and pentose [16]. MS spectral interpretation led to the identification of four quercetin (*m*/*z* 301) conjugates including quercetin-diglucoside (*m*/*z* 625.1405, C_27_H_29_O_17_, [M − H]^−^ peaks L16/18, (Figure S5A), quercetin glucoside (*m*/*z* 463.0883, C_21_H_19_O_12_, [M − H]^−^ peak L21, Figure S5B), and quercetin rhamnoside (*m*/*z* 447.0933, [M − H]^−^, C_21_H_19_O_11_, peak L23). Similarly, kaempferol aglycone (*m*/*z* 285) was detected in kaempferol-*O*-glucoside (astragalin) (*m*/*z* 447.0933, C_21_H_19_O_11_, [M − H]^−^ L22, Supplement Figure S5C) and isorhamnetin, a methylated derivative of quercetin, was identified as an aglycone in peak L38 with [M − H]^−^ of 315.051. Other characteristic fragments of quercetin and kaempferol are those at *m*/*z* 151 and 179 corresponding respectively to the A^–^ ring fragment released after RDA fission (Figure S5D) and confirming the agylcone structure in the respective flavonol peaks. In contrast, isorhamnetin, exhibited the loss of methyl from (−15 Da), (Figure S5E). Both flavonol peaks L22 and L23 are for the first time reported in *Allium*. It should be noted that no peaks for anthocyanins in either negative or positive modes were detected in *A. cepa* red cv. extract which could be attributed to the level of detection or the analysis protocol.

#### 2.3.3. Identification of Fatty Acids and Oxylipins

MS spectra of several fatty acids eluting mostly in the late elution part of the chromatogram (*R^t^* 400–600 s), were identified including linoleic (L48), palmitic acid (L49), oleic acid (L50), and stearic acid (L51) from their respective molecular ion masses at *m*/*z* 279.2324 (C_18_H_31_O_2_)^−^, *m*/*z* 255.2329 (C_16_H_31_O_2_)^−^, 281.2485 (C_18_H_33_O_2_)^−^, and 283.2638 (C_18_H_35_O_2_)^−^. A few hydroxylated fatty acids were identified from extra loss of water molecule (−18 amu) in peak (L34) [*m*/*z* 305.0709, (C_12_H_17_O_7_S)^−^], peak (L39) [*m*/*z* 329.2337, (C_18_H_33_O_5_)^−^], and peak (L47) [*m*/*z* 295.229, (C_18_H_31_O_3_)^−^]. A novel hydroxylated fatty acid present in both *Allium* species was annotated as 9,12,13-trihydroxy octadeca-7-enoic acid (*m*/*z* 329.232, [M − H]^−^, L39) based on fragment masses at *m*/*z* 311, 229, and 171, later resulting from cleavage at the C9 position, (Supplement Figure S6). Hydroxy fatty acids are recognized for their anti-inflammatory, antimicrobial, and cytotoxic activities [17];whether they contribute to *Allium* effects has yet to be determined. A sulphated oxylipin was tentatively identified as jasmonic acid-hydroxy-*O*-sulfate in peak L34 (*m*/*z* 305.0709, C_12_H_17_O_7_S, [M − H]^−^) and exhibiting the loss of 80 amu for the sulphate moiety. Another sulphated fatty alcohol was detected in peak (42) [*m*/*z* 265.1477, (C_12_H_25_O_4_S)^−^] assigned as trimethylnonanol sulphate. Both sulphate peaks L34 and L42 are reported for the first time in *Allium* and suggest occurrence of sulphur is not limited to peptides in *Allium*.

#### 2.3.4. Identification of Phenolic Acids

*Allium* species are known to accumulate phenolic acid derivatives, i.e., caffeic and ferulic acids commonly reported in metabolite profiling studies of many plant extracts. In this study, phthalic acid (L14, *m*/*z* 165.019 [M − H]^−^), caffeic acid (L20, *m*/*z* 179.0346 [M − H]^−^), ferulic acid (L27, *m*/*z* 193.0509 [M − H]^−^) and caffeic acid dimethyl ether (L36, *m*/*z* 207.0658 [M − H]^−^) were identified. Phthalic acid volatile derivatives are the major constituent of *A. atroviolaceum* [18] and its non-volatile acid form is reported for the first time in *Allium*. A peak for diethyl phthalic acid identified from GC/MS (Table 1) in commercial garlic preparation “Tomex” was also detected via UPLC/MS (L40, *m*/*z* 223.0962, C_12_H_15_O_4_ [M + H]^+^) though at a much lower response in positive ionization mode.

### 2.4. Multivariate Data Analysis of Allium Species Analysed via UPLC-MS

**Figure 4:**
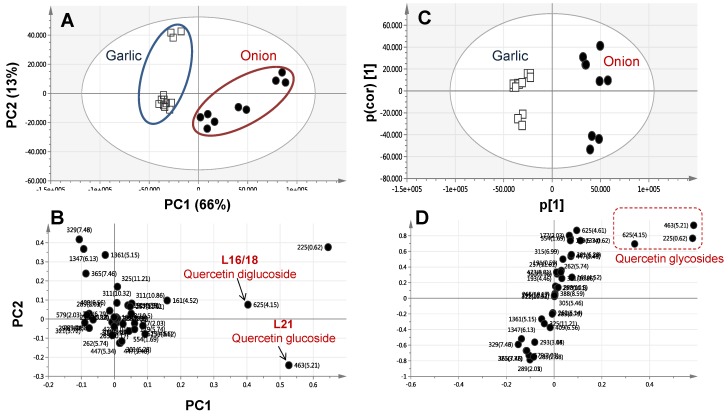
Principal component analysis (PCA) and orthogonal projection to latent structures-discriminant analysis (OPLS) supervised data analysis of modelling *A. sativum* versus *A. cepa* red cv. specimens analysed via UPLC-MS for their secondary metabolites. PCA score (**A**) and loading plot (**B**) (*n* = 3); OPLS-DA score plot (**C**) and loading S-plot (**D**). Segregation in both score plots shows enrichment of sulphur compounds in *A. sativum* versus flavonoids in *A. cepa* red cv. Peak numbering follow that listed in (Table 2) for metabolite identification via UPLC-MS.

A PCA model was constructed initially for classifying all *Allium* specimens based on metabolites analysed via UPLC-MS as data matrix. The mass signals abundance extracted from the UPLC–MS data for the seven *Allium* specimens was subjected to PCA analysis (Figure 4). The main principal component (PC) was used to differentiate between samples, i.e., PC1, accounted for 66% of the variance versus 13% for PC2. Similar to GC/MS derived PCA results, the PCA score plot showed two distinct clusters, each relating to onion and garlic individual specimens (Figure 4A), suggesting that the genotype overcomes the drying effect. Segregation in the score plot was attributed to sulphur compounds enrichment in garlic versus abundance of flavonoids in onion viz., quercetin glycosidic conjugates as revealed from the PC1 loading plot (Figure 4B). In agreement with PCA results (Figure 4A), the OPLS-DA model performed by the modelling onion specimens in one group versus garlic in another class group showed a clear separation (Figure 4C). The derived S-loading plot confirmed the abundance of flavonoids i.e., quercetin glycosides in onion (Figure 4D).

**Figure 5:**
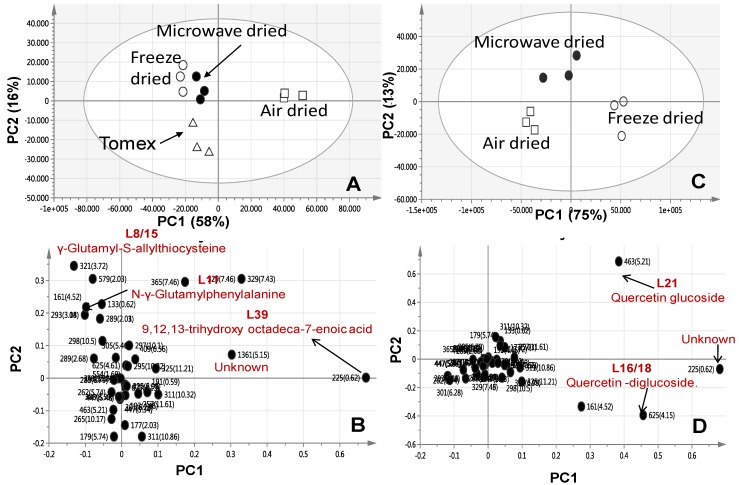
UPLC/MS based PCA score plot derived from modelling *A. sativum* specimens (**A**) and *A. cepa* red cv. specimens (**C**) one at a time separately to assess the effect of drying on metabolites composition (*n* = 3); The loading plot from *A. sativum* model (**B**) and *A. cepa* red cv. specimens (**D**) shows the most variant masses detected using UPLC/MS and contributing to the samples segregation. Metabolites are denoted with *m*/*z*/retention time (sec) pair and identifications are discussed in the text.

We further attempted to investigate the influence of the different drying methods on metabolites composition as analysed via UPLC/MS. Consequently, a PCA model was performed for freeze dried, air-dried, and microwave-dried garlic samples separately from onion. The PCA score plot (PC1 = 58% and PC2 = 16%) showed three distinct clusters, with the microwave dried garlic samples closely spaced to freeze dried ones (Figure 5A) suggesting that next to freeze drying, microwave-drying retains more secondary metabolites present in garlic. The PC1 loading plot showed an abundance of the hydroxylated fatty acid, 9,12,13-trihydroxy octadeca-7-enoic acid, in air-dried garlic samples concurrent with an enrichment of glutamyl peptides viz., γ-glutamyl-*S*-allylthiocysteine and *N*-γ-glutamyl phenylalanine in freeze and microwave dried garlic (Figure 5B).

With regards to the onion drying effect PCA model, the score plot (PC1 = 75% and PC2 = 13%) showed a comparable segregation pattern to that of garlic (Figure 5C) with the microwave dried sample positioned closer to the freeze-dried onion samples than air-dried. The loading plot confirmed the abundance of flavonoids viz. quercetin conjugates in freeze-dried onion samples compared to air-dried ones (Figure 5D).

### 2.5. Antimicrobial Activity of Allium Species

Considering the well-recognized antimicrobial activity of *Allium* [5], we found it of interest to assess how the different drying methods can impact such an effect. The in vitro minimum inhibitory concentrations (MICs) of the different dried *Allium* specimen extracts were determined against *Bacillus subtilis* growth. Results are revealed for different inhibition levels for the two different species. In particular, freeze-dried garlic extract exhibited a stronger growth inhibitory effect with a smaller IC_50_ value (IC_50_ 2.1 ± 1.3) compared to freeze-dried onion extracts (IC_50_ 5.5 ± 1.9). With regards to drying influence on garlic activity, a lower inhibition effect was observed for microwave-dried extracts (IC_50_ 4.5 ± 2.0), whereas air-dried extracts showed an IC_50_ > 50 µg/mL. Similar attenuation in the antimicrobial effect of *A. cepa* in response to drying was observed more dramatically, with no effect found for both microwave-dried and air-dried extracts. No antimicrobial effect at our assay conditions was detected for the commercially dried garlic product “Tomex”.

## 3. Discussion

Onion (*A. cepa*) and garlic (*A. sativum*) have been used for centuries either as raw vegetables for culinary purposes, or as ingredients in traditional medicine worldwide. Several processing techniques are commonly adopted to preserve food products either to increase their shelf life or to reduce some unpleasant characteristics. Nevertheless, some scientific evidence points to the fact that several biochemical modifications and inter-conversions actually occur during processing steps [1]. The current study presents an untargeted comparative metabolomics approach utilizing SPME-GC/MS and UPLC/MS high-throughput analytical technologies to provide insights onto the effect of drying protocols on the two examined *Allium* species. One commercial garlic preparation was included in this study as several garlic supplements are marketed worldwide and it is of interest to compare its composition with that of the native drug [19]. Thiosulphinates are the most examined secondary metabolite class found in the *Allium* species which originate from *S*-alk(en)yl-l-cysteine-*S*-oxide, located in the cytoplasm, through an enzymatic reaction catalysed by alliinase, a C-S lyase present in the vacuoles. In fact, thiosulphinates have been found in most *Allium* species examined so far, although with either qualitative or quantitative differences [2]. Thiosulphinates are unstable metabolites that undergo several rearrangements giving rise to an array of sulphur compounds that still exhibit biological activity including thiosulfonates, di- and tri-sulphur compounds, 2-vinyl-2,4-dihydro-1,3-dithiin, 3-vinyl-3,4- dihydro-1,2-dithiin, and ajoene [2], some of which were detected herein using GC/MS (Table 1). Consistent with our findings, Mondy et al. previously reported on sulphur compounds in *Allium* species analysed using GC/MS and in comparison to HPLC-UV. UPLC/MS was found more suited for sulphur metabolites profiling as substantial degradation of sulphur conjugates i.e., dipeptides occurred during GC/MS analysis (Table 1) [2]. While GC/MS is of great value in analysis of volatiles of moderate thermal stability, thiosulphinates from the *Allium* species are known to decompose upon heating during GC analysis. For example, Block et al. showed that bis(1-propenyl)disulphide, a common component of *Allium* distilled oil, rearranges at 85 °C to 2-mercapto-3,4-dimethyl-2,3-dihydrothiophene, leading to 3,4-dimethyl-2-thienyl disulphides. It should be noted that in our volatiles collection protocol, SPME was carried out at ambient temperature suggesting that rearrangement of sulphur compounds occurred during the GC/MS analysis of volatiles and not during the collection step. Compared to GC/MS, UPLC/MS presents a better platform for profiling the *Allium* species and to further assess the drying impact on its metabolome [20]. Not only, was a much broader metabolite class unravelled including flavonoids, fatty acids, and sulphur compounds but additionally several novel metabolites were identified in *Allium*. In particular, UPLC/MS led to the identification of two non-sulphur containing dipeptides namely, *N*-(β-hexosyl)-γ-glutamylisoleucine (L9) in *A. cepa* and *N*-(β-hexosyl)-glutamylphenylalanine (L12) in addition to a novel hydroxylated fatty acid identified as 9,12,13-trihydroxy-octadeca-7-enoic acid (L39). Furthermore, a sulphated oxylipin tentatively identified as jasmonic acid hydroxy-*O*-sulphate (L34) and trimethyl-nonanol sulphate (L42). Both sulphated lipids are reported for the first time in *Allium* species, suggesting that in *Allium*, occurrence of sulphur compounds is not restricted to peptides. In fact, the full complement of bioactive compound(s) in *Allium* species has yet to be elucidated, a necessary step to better explain its medicinal use or food properties.

Analysis of both *Allium* species via UPLC/MS (Figure 4A) showed that onion encompasses higher levels of flavonoids compared to garlic. Such a finding is consistent with the fact that onion is regarded as one of the major sources of dietary flavonoids [21]. Two major components, quercetin glucoside and quercetin di-glucoside accounted for 80% of the total flavonoids in onions. It was reported that quercetin-3,4′-*O*-glucoside and quercetin monoglucoside (quercetin 4′-*O*-glucoside) are the major flavonols in onion [22]. Flavonoids are considered important factors in the overall antioxidant activity of dietary plants [23]. In particular, quercetin exhibits anti-HIV property and protects LDL cholesterol from oxidation ultimately reducing the risk of cardiovascular diseases [2].

With regards to *A. sativum* the commercial product examined herein, our data revealed that its chemical composition varied to a large extent when compared to raw garlic (Figure 1), mostly attributed to the presence of diethyl phthalate. Phthalate esters were detected in several over-the-counter medicines from China [24] and their presence is likely to be derived either from gastroresistant film coatings, plastic packing materials, or the *Allium* plant itself. Phthalic acid volatile derivatives are major constituents of *A. atroviolaceum* [18] and the non-volatile acid form i.e. phthalic acid (L**14**) was also identified via UPLC-MS analysis (Table 2). The fact that SPME involves no solvent extraction step rules out solvent contamination during sample preparation especially as it was not detected in any of the other *Allium* specimens analysed under the same conditions.

Drying functional foods is one of the most expensive and critical processes. The cost varies depending on the employed drying method which must generally be of high efficiency and low cost. In this context, the influence of drying on *A. sativum* and *A. cepa* metabolism was further assessed using GC/MS and UPLC/MS. Considering that sulphur *Allium* metabolites are thermolabile, it is expected that they can be transformed in response to the different processing steps [1]. Consistent with such a hypothesis, air-dried and microwave-dried garlic samples analysed using GC/MS were found to group together and separately from freeze dried samples (Figure 2A) suggesting degradation of the sulphur molecules to the same extent. In contrast, a freeze dried garlic sample conducted at a much lower temperatures ca. −50 °C was more enriched in sulphur compounds namely, diallyl disulphide, diallyl trisulphide, and 3-vinyl-1,2-dithiacyclohex-5-ene (Figure 2B). Such metabolite alteration is likely to affect the garlic flavour and or bioactivity.

Some discrepancy in assessing the drying effect on garlic (Figure 2A) versus onion (Figure 2C) was observed from each respective PCA plot derived from the GC/MS dataset. Whereas air-drying produced a pronounced effect on *A. cepa*, microwave-drying retained most of the compounds still present in the freeze dried ones. PCA analysis revealed that air-dried *A. cepa* exhibited higher 2-acetylpyrrole, methyl pentyl disulphide, and allyl methyl trisulphide levels (Figure 2D). Thiosulphinates in onions are affected by heating, however, to an extent that varies depending upon the processing type [25]. In contrast, examination of the drying effect on both *Allium* drugs using the UPLC/MS platform revealed that both freeze and microwave-drying retained most of the compounds present in *A. sativum* (Figure 5A) and *A. cepa* (Figure 5C) as evident by their closer clustering along the PC1. Two glutamyl peptides namely, *N*-γ-glutamyl phenylalanine and γ-glutamyl-*S*-allyl thiocysteine were the most representative components in freeze dried and microwave-dried garlic samples (Figure 5B) compared to air-dried ones. These glutamyl peptides are considered to be storage products for nitrogen and sulphur with restricted occurrence in other plants [26]. Therefore, it was concluded that freeze drying followed by microwave-drying is less detrimental when compared to the air-drying method, likely due to the rapidness and reduced time of drying compared to air drying.

The influence of air- and microwave-drying on the flavonoid content of onion was also revealed from the UPLC/MS derived model (Figure 5C). Notably, freeze dried *A. cepa* exhibited higher quercetin conjugate content compared to the air and microwave-dried samples (Figure 5D). The observed degradation of flavonoid glycosides upon drying of samples is in agreement with previous reports showing that quercetin glycosides are degraded in oven-dried samples suggestive of plausible degradation of the flavonoid glycosides [27]. Also of note was the change in *A. sativum* fatty acid content upon sun-drying as evidenced in the detected hydroxylated fatty acid, 9,12,13-trihydroxy octadeca-7-enoic acid, first time reported in garlic (Table 2, Figure 5C). Hydroxy fatty acid derivatives are receiving increasing attention due to their anti-inflammatory, antimicrobial, and cytotoxic activities [28] as well as contributing to *Allium* effects has yet to be determined.

The biological activity of *Allium* species is also known to vary in response to various processing methods. Lemar et al. [29] reported that fresh garlic extract exhibited a stronger anti-candida effect than dried garlic powder extracts. Moreover, other scientific evidence points to the fact that fresh garlic extracts should be preferably used and are widely recommended in cases of microbial infections, as well as those derived from aqueous extracts, since the most prominent effects were achieved by using these forms of garlic preparations [1]. Such findings were also confirmed in the current study, with freeze dried garlic samples which most closely mimic fresh cloves possessing the strongest antibacterial activity against *Bacillus subtilis*, whereas air-dried garlic showed the lowest inhibition and with no effect obtained for the commercial product “Tomex”. The decrease in *A. sativum* anti-microbial activity upon drying is attributed to the thermal instability of sulphur compounds mediating its bacteriostatic action. This is supported by the observation that storage at room temperature affects antibacterial effectiveness of garlic extract and is less pronounced if stored at 0–4 °C [30]. Compared to garlic, freeze-dried onion possessed an attenuated inhibitory response towards *B. subtilis* likely as the former contains higher levels of sulphur compounds (Figure 1A). Moreover, microwave and air-dried onion extracts failed to exert a bacteriostatic effect against the tested microorganism.

## 4. Materials and Methods

### 4.1. Plant Material

*Allium sativum* and *A. cepa* red cv. bulbs were collected fresh from the field at Siwa Oasis, Egypt during the month of May 2016. Samples were cleaned, peeled, and sliced into 3 cm pieces before drying. Both sliced garlic and onion samples separately were divided into three parts and dried to 8–10% moisture content using three drying methods as follows: (a) shade drying at average temperature set at 30 °C; (b) microwave-drying by placing the plant material in a microwave with the power strengths adjusted to 1000 W (M1000) or (c) freeze drying overnight set at −50 °C. The cloves were stored at −20 °C until analysis. Commercial garlic tablets under the trade name of “Tomex” were purchased from Sekem Drug Company, Egypt. Three biological replicates were analysed for each sample.

### 4.2. Chemicals and Fibers

SPME fiber of stableflex coated with divinylbenzene/carboxen/polydimethylsiloxane (DVB/CAR/PDMS, 50/30 µm) was purchased from Supelco (Oakville, ON, Canada). All chemicals and standards were purchased from Sigma Aldrich (St. Louis, MO, USA). Acetonitrile and formic acid (LC–MS grade) were obtained from J.T. Baker (Deventer, The Netherlands), milliQ water was used for UPLC/PDA/ESI–qTOF-MS analysis.

### 4.3. Headspace Volatiles Analysis of A. sativum and A. cepa Bulbs

The HS-SPME volatile analysis was carried out as stated in [31] with slight modifications. Dried finely ground peeled bulbs (100 mg) were placed in solid phase microextraction (SPME) screw cap vials (1.5 mL) and spiked with (*Z*)-3-hexneyl acetate dissolved in water at a final concentration of 2 µg per vial. The SPME fiber was inserted manually into a vial containing seeds placed in an oven kept at 50 °C for 30 min. The fiber was subsequently withdrawn into the needle and then injected into the injection port of the gas chromatography-mass spectrometer (GC-MS). GC-Ms analysis was performed on a Schimadzu GC-17A gas chromatogram (Schimadzu, Tokyo, Japan) equipped with DB-5 column (30 m × 0.25 mm i.d. × 0.25 µm film thickness; Supelco) and coupled to Schimadzu QP5050A mass spectrometer. The interface and the injector tempreatures were both set at 220 °C. The following gradient temperature program was used for volatiles analysis. The oven temperature was kept first at 40 °C for 3 min, then increased to 180 °C at a rate of 12 °C min^−1^, kept at 180 °C for 5 min, and finally ramped at a rate of 40 °C min^−1^ to 240 °C and kept at this temperature for 5 min. The carrier gas helium was used at a total flow rate of 0.9 mL/min. Splitless injection mode was used for analysis considering the lower levels of volatiles in samples. SPME fiber was prepared for the next analysis by placing it in the injection port for 2 min at 220 °C to ensure complete elution of volatiles. Blank runs were made during sample analyses. The HP quadruple mass spectrometer was operated in EI mode at 70 eV. A scan range was set at *m*/*z* 40–500.

Volatile components were identified by comparing their retention indices (RI) relative to n-alkanes (C6–C20), mass matching to NIST, WILEY library database and with standards whenever available. Peaks were first deconvoluted using AMDIS software (National Institute of Standards and Technology, Gaithersburg, MD, USA) prior to mass spectral matching.

### 4.4. GC–MS Data Processing for Multivariate Analysis

Volatiles abundance data were prepared for multivariate data analysis by extraction using MET-IDEA software [32] for data extraction. Data were then subjected to principal component analysis (PCA) and partial least squares-discriminant analysis (OPLS-DA) using SIMCA-P version 13.0 software package (Umetrics, Umeå, Sweden).

### 4.5. Extraction Procedure and Sample Preparation for UPLC/PDA/ESI–MS Analyses and Antimicrobial Assay

Allium peeled bulbs were ground separately in a mortar with liquid nitrogen. The powder (150 mg) was then homogenized with 6 mL MeOH containing 5 μg/mL umbelliferone (internal standard) using a Turrax mixer (11,000 rpm) for five 20 s periods. To prevent heating, a period of 1 min separated each mixing period. Extracts were then vortexed vigorously and centrifuged at 3000 g for 30 min to remove plant debris. An amount of 3 μL of the supernatant was used for UPLC/PDA/ESI–ion trap MS analysis. Chromatographic conditions and mass spectrometer parameters follow that described in [18,20].

### 4.6. UPLC-Orbitrap HRMS Analysis

The negative and positive ion high-resolution ESI and collision-induced dissociation (CID) MSn spectra were obtained from an Orbitrap Elite mass spectrometer (Thermo Fisher Scientific, Darmstadt, Germany) equipped with a heated electrospray ion source (negative spray voltage of 3 kV, positive 4 kV, capillary temperature of 300 °C, source heater temperature of 250 °C, FTMS resolution of 30.000). Nitrogen was used as sheath and auxiliary gas. The MS system was coupled to an UHPLC system (Dionex UltiMate 3000, Thermo Fisher Scientific), equipped with a RP-18 column (particle size 1.8 µm, pore size 100 Å, 150 mm × 1 mm ID, Acquity HSS T3, Waters; column temperature of 40 °C) and a photodiode array detector (220–600 nm, Thermo Fisher Scientific). The mobile phases were H_2_O (A; Fluka Analytical, LC-MS Chromasolv) and CH_3_CN (B; Fluka Analytical, LC-MS Chromasolv) with 0.1% formic acid, using the following binary gradient at a flow rate of 150 μL/min: 0 to 1 min, isocratic 95% A, 5% B; 1 to 11 min, linear from 5% to 100% B; 11 to 19 min, isocratic 100% B; and 19 to 30 min, isocratic 5% B. The injection volume was 2 μL. The CID mass spectra (buffer gas: helium) were recorded using a normalized collision energy (NCE) of 35%. The instrument was externally calibrated by the Pierce ESI negative ion calibration solution (product No. 88324) and Pierce ESI positive ion calibration solution (product No. 88323) from Thermo Fisher Scientific. The data were evaluated using the software Xcalibur 2.2 SP1. Metabolites were characterized by their UV–VIS spectra (220–600 nm), retention times relative to external standards, mass spectra and comparison to phytochemical dictionary of natural products database (CRC) and reference literature.

### 4.7. UPLC/MS Data Processing for Multivariate Analysis

Relative quantification of *Allium* metabolites analysed after UHPLC–MS was performed using XCMS data analysis software, which can be downloaded freely as an R package from the Metlin Metabolite Database (The Scripps Research Institute, La Jolla, CA, USA). Data were subjected to PCA and OPLS-DA, using the SIMCA-P 13.0 software package (Umetrics, Umea, Sweden). Markers were subsequently identified by analyzing the S-plot, which was declared with covariance (p) and correlation (pcor). All variables were mean centred and scaled to Pareto variance.

### 4.8. Antimicrobial Effect Determined Using Minimum Inhibitory Concentration (MIC)

The minimum inhibitory concentration (MIC) was determined using a turbidity method modified from an earlier described protocol [33]. Dried methanol extracts for *A. cepa* or *A. sativum* were weighed at a concentration of 1000 µg/mL in DMSO and kept at −80 °C until further use. *Bacillus subtilis* strain ATCC 6051 was cultured for 24 h on agar plates supplemented with RPMI 1640 media in a 37 °C incubator. The bacteria were re-incubated with fresh media prior to further agitation for 16 h. The optical density was monitored at 612 nm and a serial dilution was performed. The bacteria were transferred into 96-well microtiter plates prepared in final concentrations of 10 and 100 µg/mL volume of *Allium* extracts. The experiments were carried out in triplicate and the optical density in each well was quantified using a microplate reader set at 600 nm (Beckman Coulter, DTX 880 Multimode Reader, Krefeld, Germany).

## 5. Conclusions

The present study aimed to investigate the chemical composition of onion and garlic via SPME-GC/MS and UPLC/MS and further to use both platforms to assess the drying impact on the composition of metabolites as monitored by each technology platform. Several sulphur degradation products were monitored via SPME-GC/MS likely due the thermolabile nature of *Allium* thiosulphinates, whereas UPLC-MS provided better coverage of the sulphur compounds and other metabolite classes viz., flavonoids and phenolic acids. Further investigation of the influence of different drying methods in both *Allium* species revealed that freeze and microwave-drying retained more of the compounds present in garlic. Two glutamyl peptides namely, *N*-γ-glutamyl phenylalanine and γ-glutamyl-S-allyl thiocysteine were the most representative components in freeze and microwave-dried garlic samples. Similarly, the flavonoid content of onion was significantly altered post drying, remaining more abundant in freeze-dried compared to air- and microwave-dried samples. Despite the advantage of SPME GC/MS in unravelling a phthalate ester “diethyl-phthalate” in commercial garlic preparation that could affect product overall quality, it showed inconsistent modelling results for the drying impact on *Allium* compared to UPLC-MS. Metabolites analyses using either technique clearly showed that species-based separation predominates over the drying effect as revealed from their respective PCA score plot. Antimicrobial analyses of onion and garlic against *B. subtilis* showed different inhibition levels, with freeze dried garlic extracts exhibiting higher inhibitory responses towards the *B. subtilis* compared to that of onion, likely due to its enrichment in sulphur compounds.

We acknowledge that our selection of *Allium* resources does not cover all worldwide variations, but our approach is certainly feasible for analysing *Allium* samples either raw or in different formulations from further sources. The same workflow of sample preparation, measurement and processing can indeed be easily transferred to investigate other processing factors such as storage, harvesting time and/or seasonal variation impact on *Allium* secondary metabolites composition.

## Figures and Tables

**Table 1:** Volatiles identified via solid-phase microextraction coupled to gas chromatography mass spectrometry (SPME-GC/MS) analysis of *A. sativum* and *A. cepa* red cv. and with amounts expressed as relative percentile (%), *n* = 3. Retention index (RI) is calculated relative to an alkane series C6–C20 analysed under the same conditions.

Peak No.	r.t. (min)	RI	Volatiles	Relative Abundance (%)						
				*A. sativum*				*A. cepa* Red cv.		
				Fresh	Sun-Dried	Microwave-Dried	Tomex	Fresh	Sun-Dried	Microwave-Dried
**M1**	5.867	847	Diallyl sulphide	tr.	0.05	0.53	tr.	-	-	-
**M2**	7.442	924	1-propenyl methyl disulphide	-	-	-	-	5.27	5.32	4.41
**M3**	7.986	958	Dimethyl trisulphide	-	-	-	-	6.09	6.46	4.35
**M4**	8.003	969	Dimethyl trisulphide isomer	tr.	tr.	0.03	tr.	-	-	-
**M5**	9.792	1068	2-Acetylpyrrole	-	-	-	-	0.45	12.21	1.94
**M6**	9.85	1074	Diallyl disulphide	45.99	99.11	46.98	0.08	-	-	-
**M7**	10.167	1092	Tetramethylpyrazine	0.10	tr.	3.35	tr.	-	-	-
**M8**	10.243	1097	2-Propenylthioacetonitrile	1.27	tr.	0.5	tr.	-	-	-
**M9**	10.307	1101	Isopropyl-α-mercaptopropionate	-	-	-	-	9.5	1.52	3.51
**M10**	10.717	1131	Allyl methyl trisulphide	0.43	tr.	1.43	0.04	-	-	-
**M11**	10.892	1143	Diethanol disulphide	-	-	-	-	8.93	5.22	4.29
**M12**	10.99	1149	Methyl pentyl disulphide	-	-	-	-	0.48	18.67	7.32
**M13**	11.046	1153	*cis*-Methyl propenyl sulphide	0.43	tr.	0.02	0.007	-	-	-
**M14**	11.107	1157	Allyl methyl trisulphide	-	-	-	-	13.98	18.84	19.43
**M15**	11.118	1159	Unknown sulphur	tr.	0.004	0.24	0.0002	-	-	-
**M16**	11.157	1162	Geranyl nitrile	tr.	0.003	0.07	tr.	-	-	-
**M17**	11.357	1175	Methyl 2-methylheptanoate	tr.	tr.	0.02	tr.	-	-	-
**M18**	11.5	1186	3-Ethenyl-1,2-dithi-4-ene	0.02	0.008	0.28	0.0005	-	-	-
**M19**	11.517	1189	Diallyl disulphide isomer	0.04	0.01	0.47	0.001	-	-	-
**M20**	11.751	1203	Unknown sulphur	0.01	0.01	0.906	0.001	-	-	-
**M21**	11.86	1212	3-Vinyl-1,2-dithiacyclohex-5-ene	31.8	0.19	4.306	0.007	-	-	-
**M22**	11.873	1212	3-Ethenyl-1,2-dithi-5-ene isomer	0.04	0.03	0.85	0.001	-	-	-
**M23**	11.875	1212	Dimethyl tetrasulphide	-	-	-	-	3.51	2.73	1.98
**M24**	12.158	1234	Unknown	-	-	-	-	0.43	2.11	1.64
**M25**	12.161	1234	*p*-Cuminaldehyde	tr.	0.002	0.03	tr.	-	-	-
**M26**	12.182	1236	3-Isopropyl benzaldehyde	tr.	0.002	0.106	tr.	-	-	-
**M27**	12.317	1246	4,7-Dimethylundecane	-	-	-	-	0.59	1.14	1.91
**M28**	12.917	1293	Diallyl trisulphide	19.87	0.32	28.86	0.06	-	-	-
**M29**	12.919	1292	(Allylsulfanyl)acetonitrile	-	-	-	-	3.24	0.34	4.52
**M30**	13.23	1316	Dipropyl trisulphide	-	-	-	-	3.75	3.51	5.108
**M31**	13.37	1328	unknown sulphur	-	-	-	-	17.06	9.18	21.01
**M32**	13.42	1332	Diallyl trisulphide isomer	-	-	-	-	3.85	2.69	11.45
**M33**	14.00	1381	4-(Methylsulfinyl)butanenitrile	0.002	0.02	2.27	0.003	-	-	-
**M34**	14.033	1382	Unknown sulphur	0.002	0.012	1.17	0.002	-	-	-
**M35**	15.408	1495	unknown hydrocarbon	-	-	-	-	1.56	2.19	2.37
**M36**	15.98	1535	Diallyl tetrasulphide	0.03	0.11	7.32	0.014	-	-	-
**M37**	16.18	1549	2,4-Dimethyl-5,6-dithia-2,7-nonadienal	tr.	0.002	0.03	tr.	-	-	-
**M38**	16.183	1549	Unknown	tr.	0.009	0.06	tr.	-	-	-
**M39**	16.291	1577	Ethyl dodecanoate	tr.	tr.	0.027	0.003	-	-	-
**M40**	16.717	1597	Diethyl phthalate	tr.	tr.	0.03	99.75	-	-	-
**M41**	17.00	1605	2,4-Dimethyl-5,6-dithia-2,7-nonadienal	-	-	-	-	21.24	7.8	4.68
**M42**	18.058	1661	Unknown sulphur	tr.	0.031	0.04	tr.	-	-	-

**Table 2:** Metabolites identified via UPLC/PDA/orbitrap-MS in methanol extracts of *A. sativum* and *A. cepa* red cv. extracts using negative and positive ionization mode.

Peak	Rt Sec	MS	UV nm	Formula	Error ppm	MS/MS	Metabolite	Class	*A. sativum*	*A. cepa*
**L1**	26	176.0954	265	C_6_H_10_NO_3_S	−0.3	-	Unknown	Peptide	+	-
**L2**	38	191.0196	267	C_6_H_7_O_7_	0.1	-	Citric acid/Isocitric acid	Organic acid	+	-
**L3**	70	337.1711	-	C_18_H_27_O_3_NS	1.4	319, 257, 175	Unknown	-	+	-
**L4**	86	554.1658	295	C_28_H_28_NO_11_	−2.7	392	Simmondsin-di-*O*-de-Me, di-*O*-benzoyl	Nitrile	-	+
**L5**	100	451.1401	-	C_17_H_27_O_10_N_2_S	0.7	433, 361, 289	*N*-Hexosyl-γ-glutamyl-*S*-allylcysteine	-		
**L6**	109	289.0873	-	C_11_H_17_N_2_O_5_S	−3.1	271, 215, 128	*N-γ*-Glutamyl-*S*-allylcysteine.	Peptide	+	-
**L7**	129	259.1298	281	C_11_H_19_N_2_O_5_	0.6	203	*N*-γ-Glutamylisoleucine	Peptide	-	+
**L8**	135	321.0612	-	C_11_H_17_N_2_O_5_S_2_	1.1	303, 249, 128	γ-Glutamyl-*S*-allylthiocysteine	Peptide	+	
**L9**	149	421.182	281	C_17_H_29_N_2_O_10_	1.1	403, 331, 259	*N*-Hexosyl-γ-glutamylisoleucine	Peptide	-	+
**L10**	153	289.0873	-	C_11_H_17_N_2_O_5_S	−3.1	271, 215, 128	*N-γ*-Glutamyl-*S*-allylcysteine	Peptide	+	-
**L11**	171	293.1135	218	C_14_H_17_N_2_O_5_	2.7	165	*N*-γ-Glutamylphenylalanine	Peptide	+	+
**L12**	171	455.1666	290	C_20_H_27_N_2_O_10_	1.2	437, 365, 293	*N*--Hexosyl-glutamylphenylalanine	Peptide	+	+
**L13**	184	353.0285	225, 279	C_15_H_21_N_4_O_2_S_2_	3.9	165, 121	Allithiamine	Thiamine deriv.	+	-
**L14**	184	165.019	-	C_8_H_5_O_4_	−0.6	-	Phthalic acid	Phenolic acid	+	-
**L15**	207	321.0587	-	C_11_H_17_N_2_O_5_S_2_	0.0	249, 171	γ-Glutamyl-*S*-allylthiocysteine	Peptide	+	-
**L16**	231	625.1405	266, 343	C_27_H_29_O_17_	0.2	361, 241	Quercetin-*O*-diglucoside.	Flavonol	-	+
**L17**	235	361.1081	266, 344	C_22_H_17_O_5_	0.1	241	Unknown	-	-	+
**L18**	235	625.1405	266, 343	C_27_H_29_O_17_	0.2	361, 241	Quercetin-*O*-diglucoside.	Flavonol	-	+
**L19**	256	161	360	C_9_H_6_O_3_	−0.1	-	Umbelliferone (IS)	Coumarin	-	-
**L20**	273	179.0346	279	C_9_H_7_O_4_	2.1	-	Caffeic acid	Phenolic acid	+	-
**L21**	302	463.0883	266, 365	C_21_H_19_O_12_	−0.2	301	Quercetin-*O*-glucoside	Flavonol	-	+
**L22**	309	447.0933	267, 362	C_21_H_19_O_11_	−1.0	285	Kaempferol-*O*-glucoside (Astragalin)	Flavonol	-	+
**L23**	311	447.0933	267, 362	C_21_H_19_O_11_	−1.0	301	Quercetin-*O*-rhamnoside	Flavonol	-	+
**L24**	318	477.1029	365	C_22_H_21_O_12_	2.1	315	Isorhamnetin-*O*-hexoside	Flavonol	-	+
**L25**	324	228.1241	-	C_11_H_18_NO_4_	0.3	-	Unknown	-	-	+
**L26**	330	409.091	276	C_26_H_17_O_3_S	−1.5	-	Unknown	-	+	-
**L27**	330	193.0509	276	C_10_H_9_O_4_	−1.3	-	Ferulic acid	Phenolic acid	+	-
**L28**	335	262.1089	-	C_14_H_16_NO_4_	−1.6	-	*N*-*p*-Coumaroyl-valine	Acylated amino acid	-	+
**L29**	346	238.109	296	C_12_H_16_NO_4_	0.6	164	Unknown	-	-	+
**L30**	351	262.1088	-	C_14_H_16_NO_4_	−1.1	-	*N-p*-Coumaroyl-valine isomer	Acylated amino acid	-	+
**L31**	352	273.08752	-	C_14_H_13_O_4_N_2_	0.6	229	Unknown	-	-	+
**L32**	354	419.0927	-	C_27_H_15_O_5_	−0.4	-	Unknown	-	+	-
**L33**	365	301.0357	370	C_15_H_9_O_7_	−0.8	161, 179	Quercetin	Flavonol	-	+
**L34**	372	305.0709	-	C_12_H_17_O_7_S	−2.9	287, 225	Jasmonic acid-hydroxy-*O*-sulfate	Oxylipid	+	-
**L35**	378	423.1193	-	C_22_H_19_N_2_O_7_	1.1	-	Unknown	-	+	-
**L36**	382	207.0658	-	C_11_H_11_O_4_	2.1	177	Caffeic acid dimethyl ether	Phenolic acid	+	-
**L37**	413	285.0403	-	C_15_H_9_O_6_	0.6	161, 175	Kaempferol	Flavonol	-	+
**L38**	423	315.051	-	C_16_H_11_O_7_	0.1	300, 161, 176	Isorhamnetin	Flavonol	-	+
**L39**	439	329.2337	-	C_18_H_33_O_5_	−0.4	311, 293, 257, 229, 211, 175	9,12,13-trihydroxy octadeca-7-enoic acid	Fatty acid	+	+
**L40+**	530	223.0962	-	C_12_H_15_O_4_	0.3	249	Diethylphthalate	Aromatic	+	-
**L41**	610	388.3057	-	C_21_H_42_NO_5_	3.3	249, 317	Unknown	-	-	+
**L42**	615	265.1477	-	C_12_H_25_O_4_S	0.9	175	Trimethylnonanol sulphate	Oxylipid	+	+
**L43**	641	297.15283	-	C_12_H_25_O_8_	−1.5	183	Unknown	-	+	-
**L44**	652	297.10323	-	C_19_H_21_O_3_	4.3	183	Unknown	-	-	+
**L45**	662	311.1686	-	C_20_H_23_O_3_	−5.9	-	Unknown	-	-	+
**L46**	670	311.1137	-	C_13_H_27_O_8_	−2.0	-	Unknown	-	+	+
**L47**	680	295.2276	-	C_18_H_31_O_3_	1.1	249	Oxo-octadecenoic acid	Fatty acid	+	-
**L48**	867	279.2324	-	C_18_H_31_O_2_	2.9	181	Linoleic acid	Fatty acid	+	+
**L49**	912	255.2329	-	C_16_H_31_O_2_	0.2	-	Palmitic acid	Fatty acid	+	-
**L50**	927	281.2485	-	C_18_H_33_O_2_	1.5	-	Oleic acid	Fatty acid	+	+
**L51**	983	283.2638	-	C_18_H_35_O_2_	1.5	-	Stearic acid	Fatty acid	+	+

## Supplementary Materials

Supplementary Materials are available online.
