# Supplementary material for: Phytochemical Profiles and Antimicrobial Activities of Allium cepa Red cv. and A. sativum Subjected to Different Drying Methods: A Comparative MS-Based Metabolomics
Source: Molecules. 2017 May 8;22(5):761. doi: 10.3390/molecules22050761 (PMC6154556; doi:10.3390/molecules22050761)
Supplement: Supplementary file 1 [file molecules-22-00761-s001.pdf]

Supplementary Materials: Phytochemical Profiles and Antimicrobial Activities of *Allium cepa* Red cv. and *A. sativum* Subjected to Different Drying Methods: A Comparative MS-Based Metabolomics

Mohamed A. Farag, Sara E. Ali, Rashad H. Hodaya, Hesham R. El-Seedi, Haider N. Sultani, Annegret Laub, Tarek F. Eissa, Fouad O. F. Abou-Zaid and Ludger A. Wessjohann

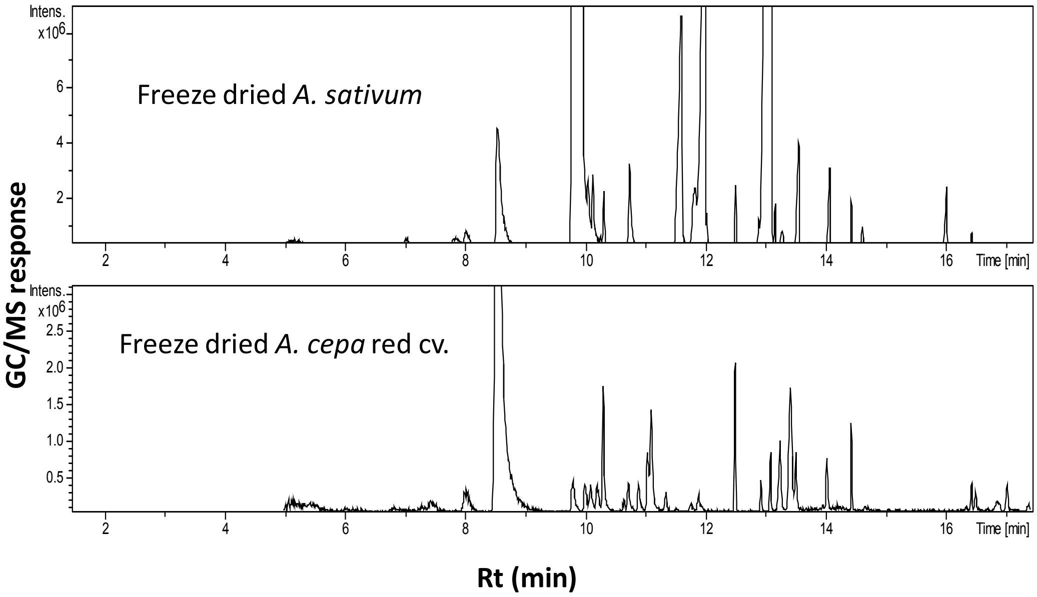

Fig. S1 Representative SPME–GC/MS chromatograms of volatiles collected from freeze dried *A. sativum* and *A. cepa* red cv.

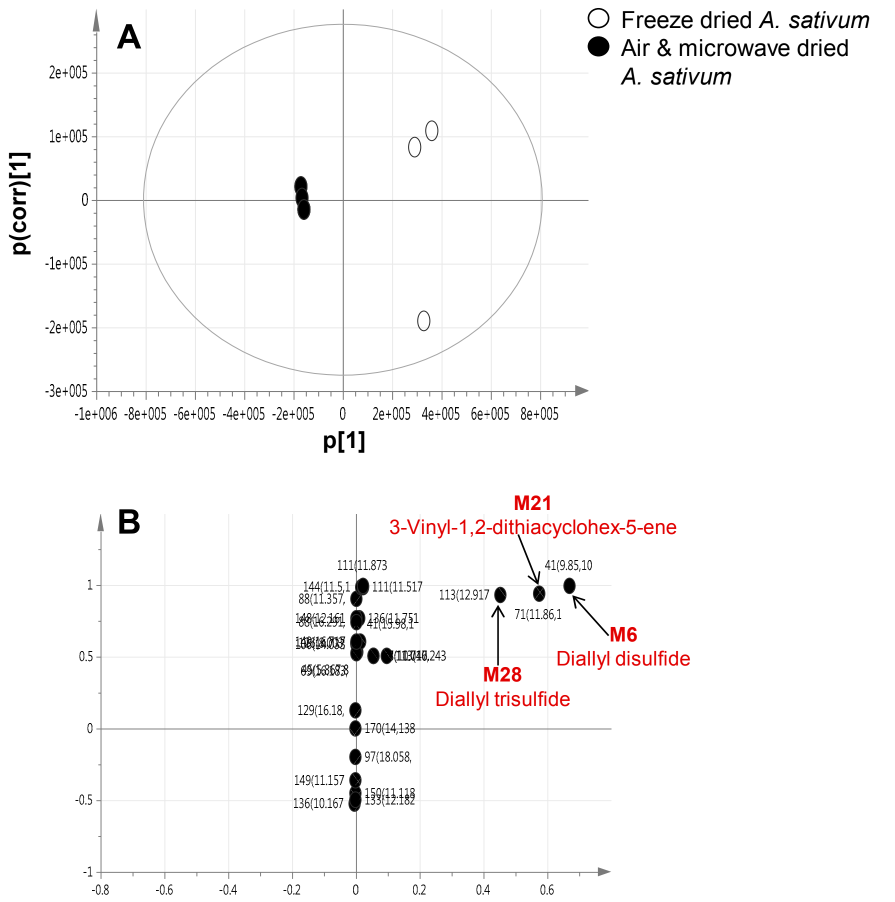

Fig. S2 SPME-GC/MS based orthogonal projection to latent structures-discriminant analysis (OPLS-DA) of freeze dried A, sativum ( ) versus air and microwave dried garlic ( ). (A) OPLS-DA score plot (B) loading plot derived from samples modeled against each other. The loading plot shows the covariance p[1] against the correlation p(corr)[1] of the variables of the discriminating component of the OPLS-DA model. Selected variables are highlighted in the loading plot with each metabolite denoted by its mass/rt (min) value: M6; diallyl disulfide, M21; 3-Vinyl-1,2-dithiacyclohex-5-ene and M28; diallyl trisulfide. Peaks numbering follow those listed in (**Table 1**) for volatiles identification using SPME-GC/MS.

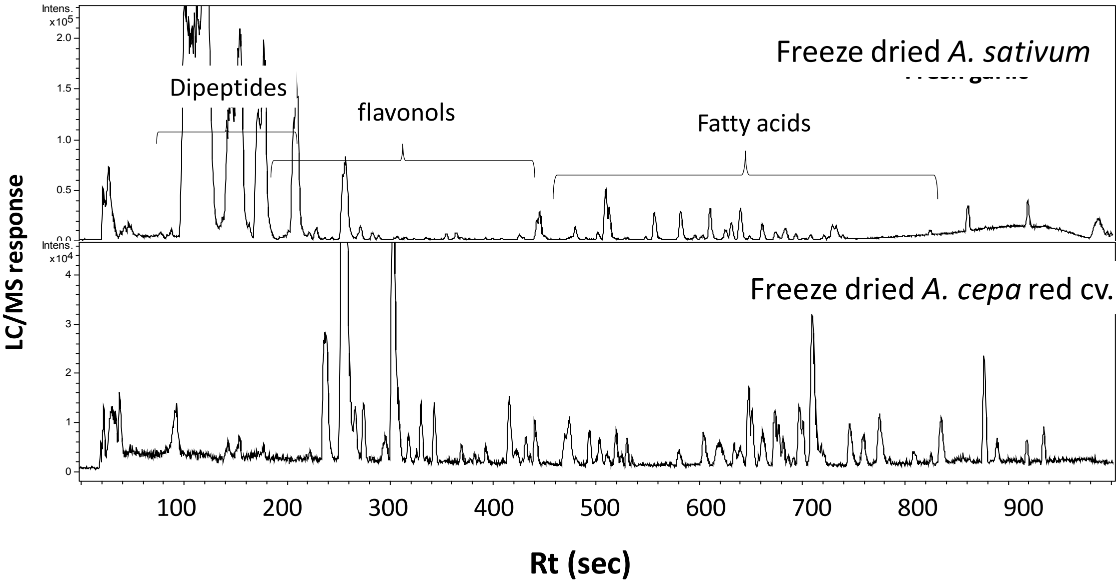

Fig. S3 Representative UPLC-QTOF-MS fingerprinting analyses of freeze dried garlic *A. sativum* and red onion cv. *A. cepa* extracts analysed using negative ionization mode.

Peak L6, N-γ-Glutamyl-S-allylcysteine, 289.0873 (M-H)^-

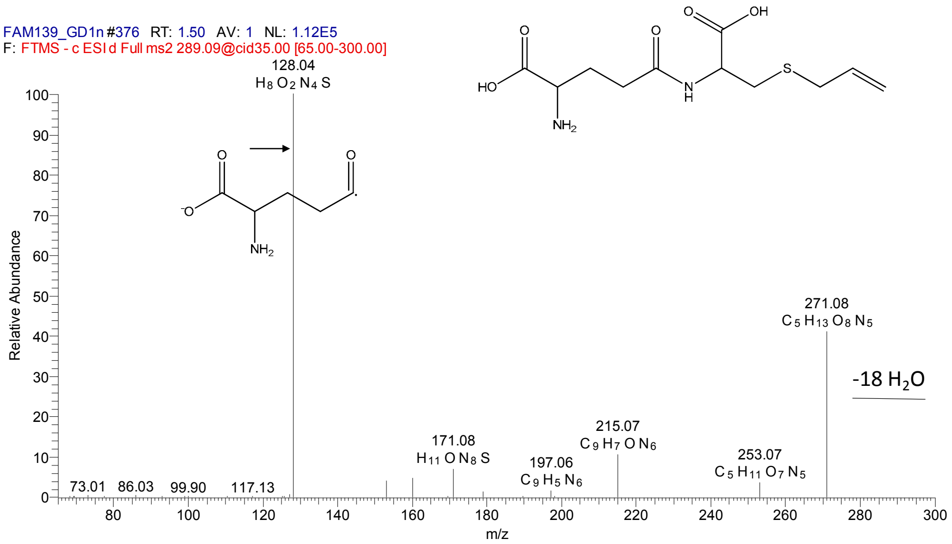

(**A**)

Peak L8, γ-Glutamyl-S-allylthiocysteine, 321.061 (M-H)-

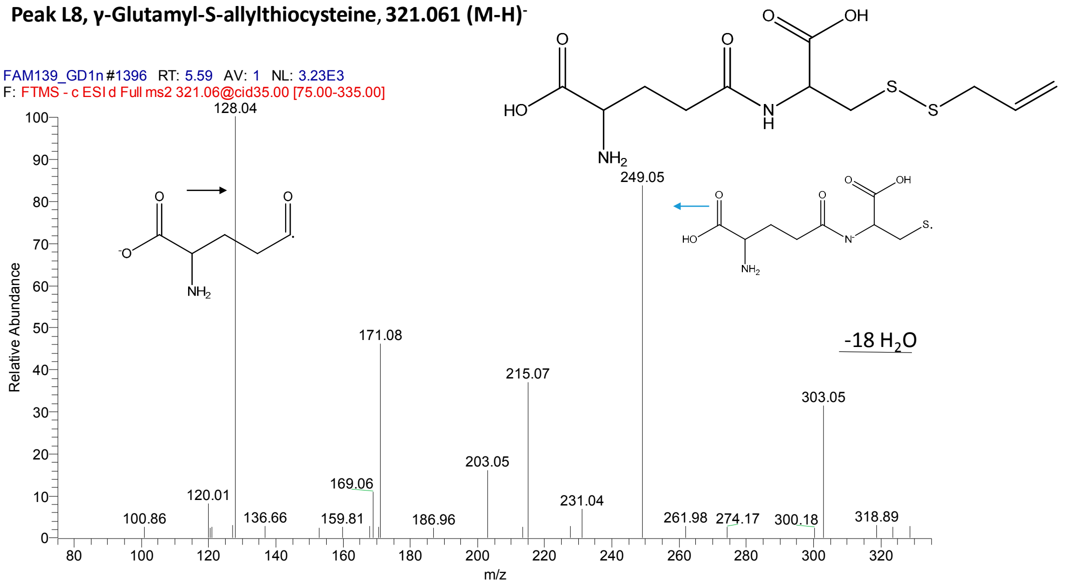

(**B**)

Peak L5, N-γ-Glutamyl-S-allylcysteine, 451.1401 (M-H)-

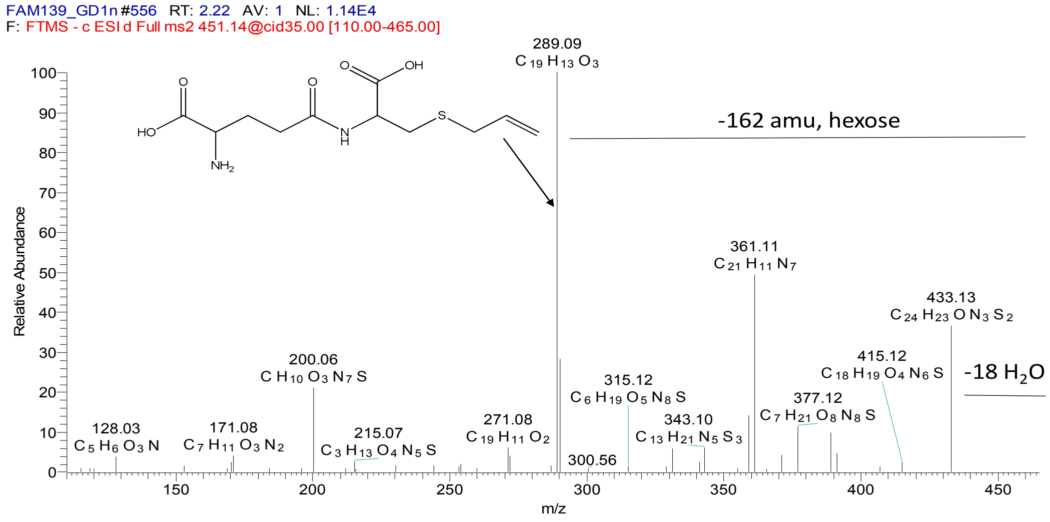

(**C**)

Peak L9, N-(β-hexosyl)-γ-Glutamylisoleucine, 421.182 (M-H)⁻

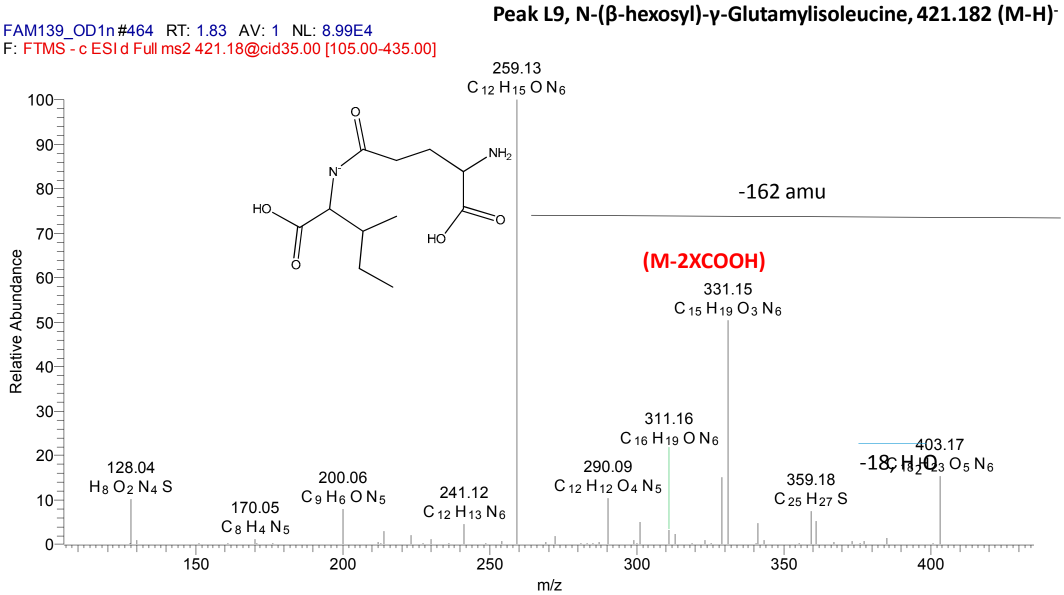

(D)

Peak L12, , N-(β-hexosyl)-Glutamylphenylalanine, 455.166 (M-H)⁻

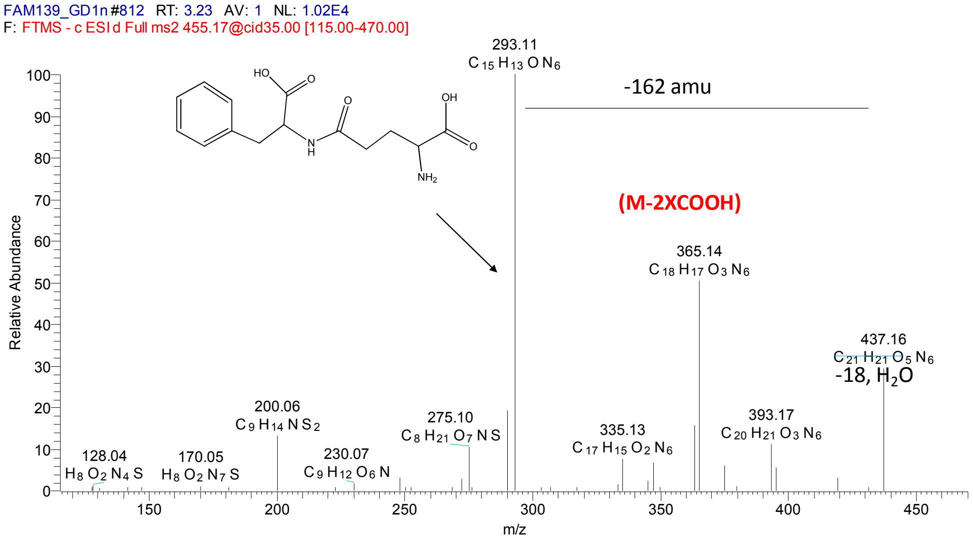

(E)

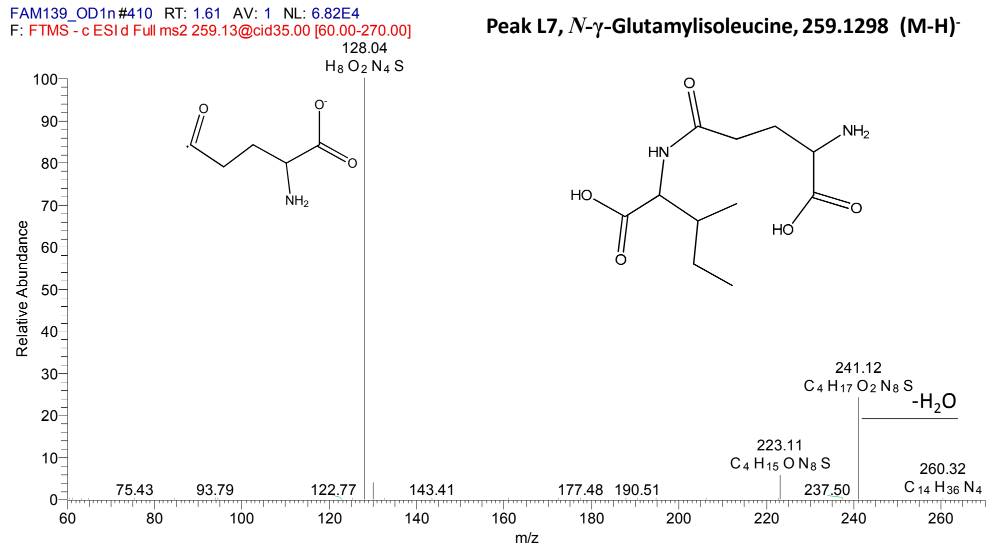
FAM139_OD1n #410 RT: 1.61 AV: 1 NL: 6.82E4
F: FTMS - c ESI d Full ms2 259.13@cid35.00 [60.00-270.00]
Peak L7, N-γ-Glutamylisoleucine, 259.1298 (M-H)-
128.04
H8 O2 N4 S
241.12
C4 H17 O2 N8 S
-H2O
223.11
C4 H15 O N8 S
260.32
C14 H36 N4
75.43
93.79
122.77
143.41
177.48
190.51
237.50
Relative Abundance
m/z

(F)

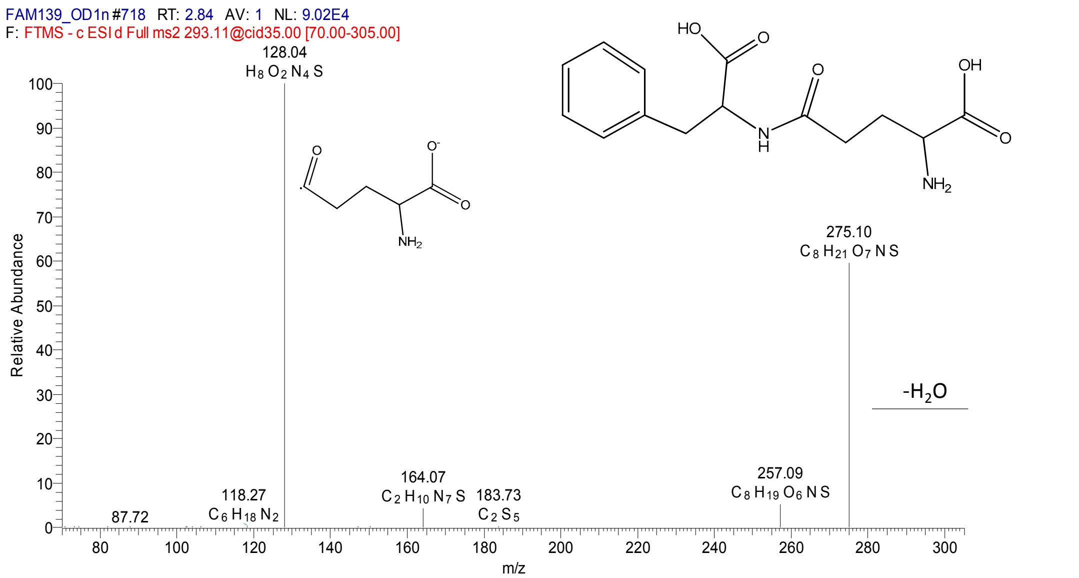
Peak L11, N-γ-Glutamylphenylalanine, 293.1135 (M-H)-
FAM139_OD1n #718 RT: 2.84 AV: 1 NL: 9.02E4
F: FTMS - c ESI d Full ms2 293.11@cid35.00 [70.00-305.00]
128.04
H8 O2 N4 S
275.10
C8 H21 O7 N S
-H2O
257.09
C8 H19 O6 N S
164.07
C2 H10 N7 S
183.73
C2 S5
118.27
C6 H18 N2
87.72
Relative Abundance
m/z

(G)

Peak 3, 337.1711 (M+H)$^+$

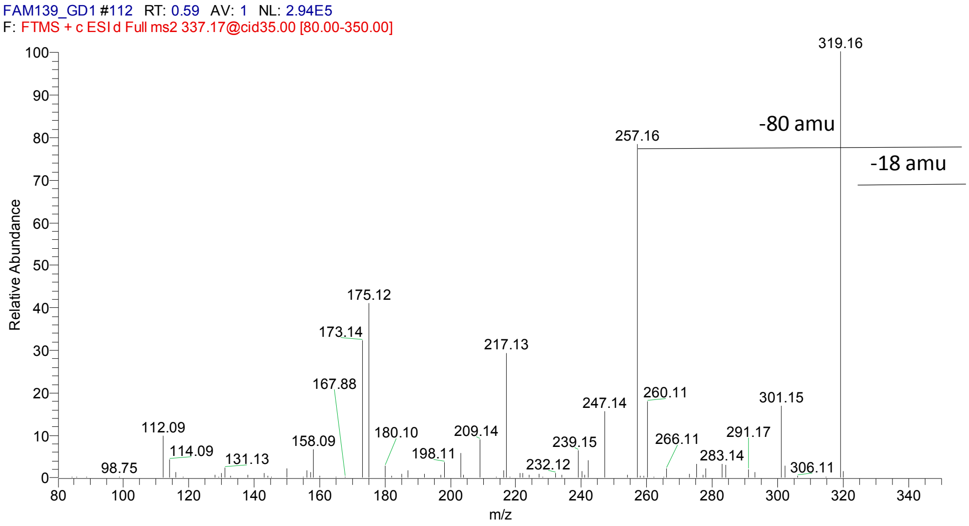

(**H**)

Fig. S4. (A) ESI-MS/MS spectrum of peak (L6) in the negative ion mode. **(B)** ESI-MS/MS spectrum of peak (8) in the negative ion mode. **(C)** ESI-MS/MS spectrum of peak (L5) in the negative ion mode. **(D)** ESI-MS/MS spectrum of peak (L9) in the negative ion mode. **(E)** ESI-MS/MS spectrum of peak (L12) in the negative ion mode. **(F)** ESI-MS/MS spectrum of peak (L7) in the negative ion mode. **(G)** ESI-MS/MS spectrum of peak (L11) in the negative ion mode. **(H)** ESI-MS/MS spectrum of peak (L3) in the negative ion mode.

Peak L16, Quercetin-*O*-diglucoside, 625.1405 (M-H)$^-$

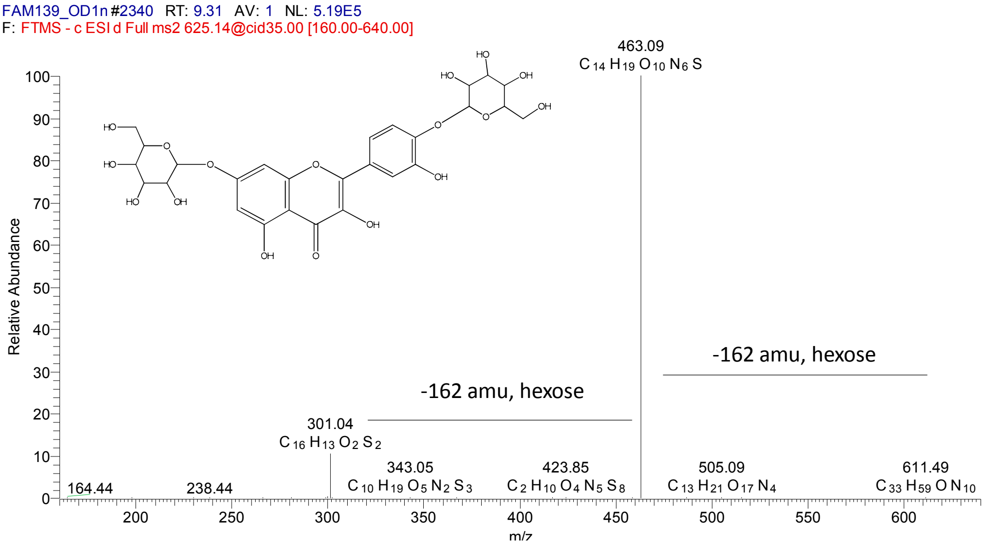

(**A**)

Peak L21 Quercetin hexoside *m/z* 463.0883 [M-H]$^-$

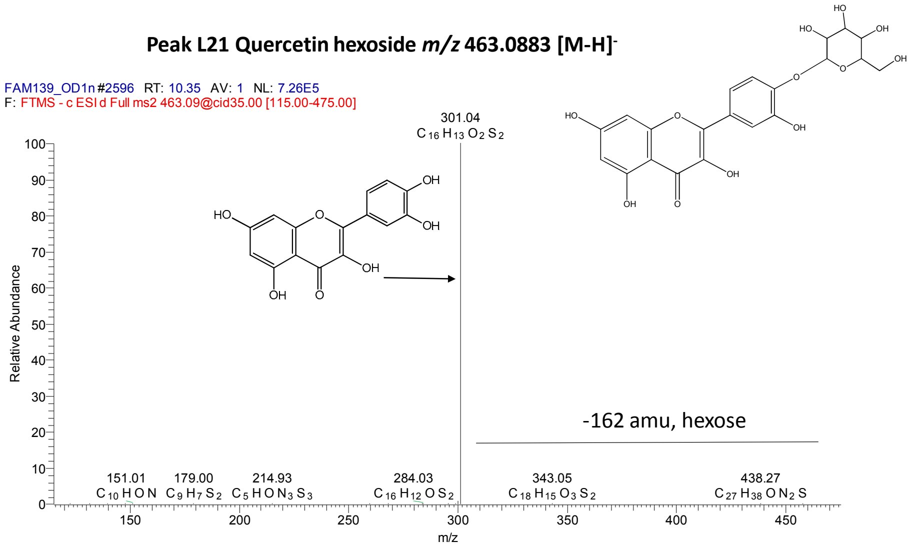

(**B**)

Peak L22, Kaempferol-*O*-hexoside (astragalin) *m/z* 447.0933 [M-H]$^-$

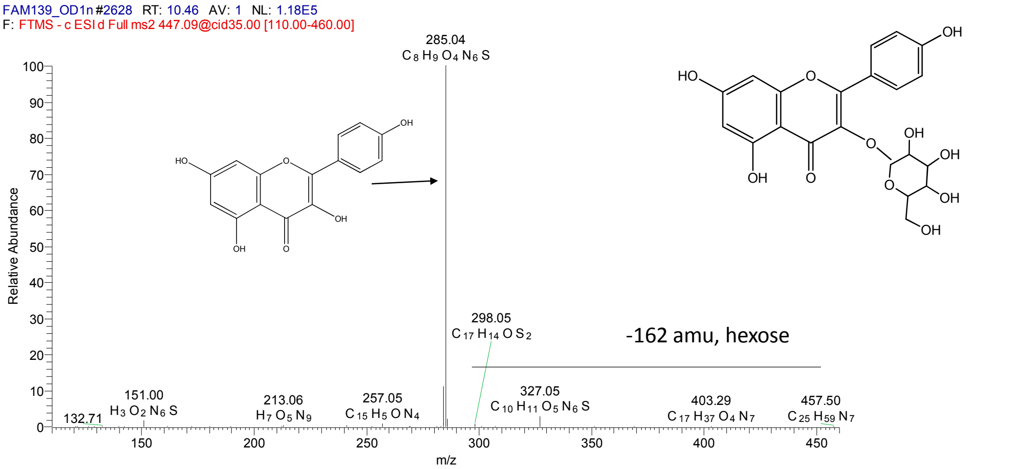

(**C**)

Peak L33, quercetin *m/z* 301.0357 [M-H]$^-$

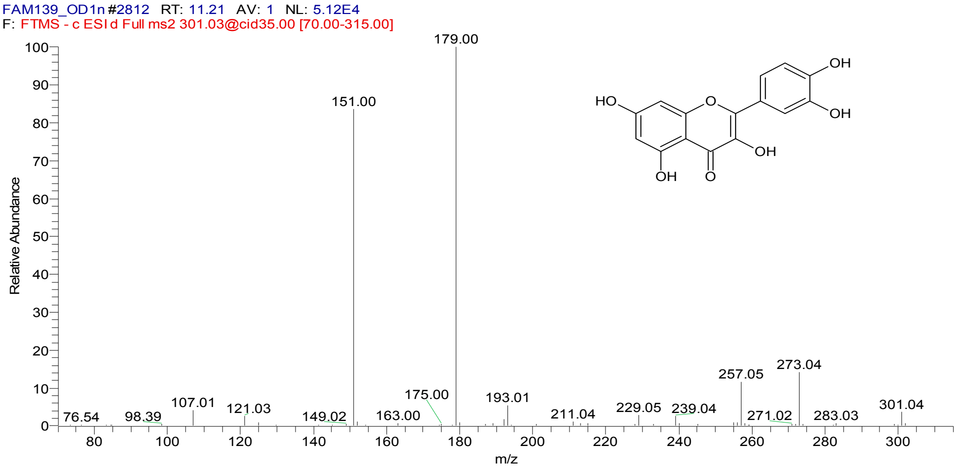

(**D**)

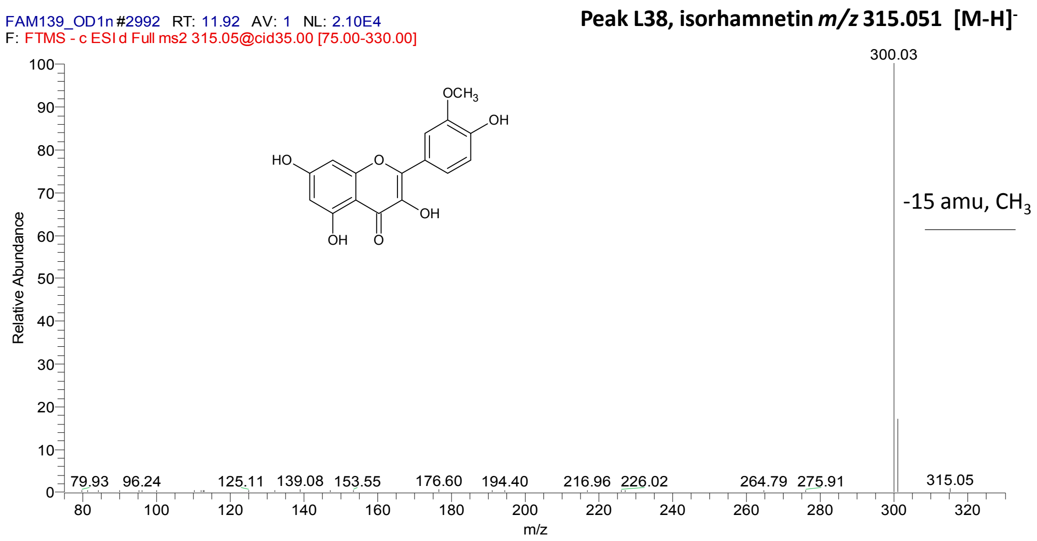

(**F**)

Fig. S5. (**A**) ESI-MS/MS spectrum of peak (L16) in the negative ion mode. (**B**) ESI-MS/MS spectrum of peak (L21) in the negative ion mode. (**C)** ESI-MS/MS spectrum of peak (L22) in the negative ion mode. (**D**) ESI-MS/MS spectrum of peak (L33) in the negative ion mode. (**E**) ESI-MS/MS spectrum of peak (L38) in the negative ion mode.

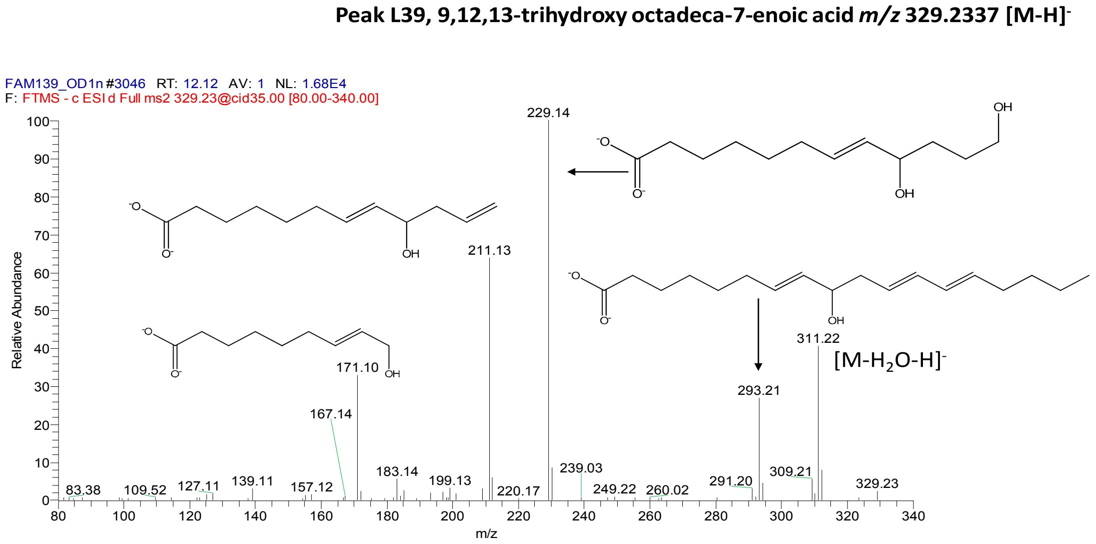

Fig. S6. ESI-MS/MS spectrum of peak (L39) in the negative ion mode.
